# Self-regulation as a resource for coping with developmental challenges during middle childhood and adolescence: the prospective longitudinal PIER_YOUTH_-study

**DOI:** 10.1186/s40359-023-01140-3

**Published:** 2023-04-03

**Authors:** P. Warschburger, M. S. Gmeiner, R. Bondü, A. M. Klein, R. Busching, B. Elsner

**Affiliations:** 1 Department of Psychology, University of Potsdam, Karl-Liebknecht-Str. 24-25, 14476 Potsdam, Germany; 2 Psychologische Hochschule Berlin, Am Köllnischen Park 2, 10179 Berlin, Germany; 3 International Psychoanalytic University Berlin, Stromstraße 1, 10555 Berlin, Germany

**Keywords:** Self-regulation, Adolescence, Prospective longitudinal study, Mental health

## Abstract

**Background:**

Self-regulation (SR) as the ability to regulate one’s own physical state, emotions, cognitions, and behavior, is considered to play a pivotal role in the concurrent and subsequent mental and physical health of an individual. Although SR skills encompass numerous sub-facets, previous research has often focused on only one or a few of these sub-facets, and only rarely on adolescence. Therefore, little is known about the development of the sub-facets, their interplay, and their specific contributions to future developmental outcomes, particularly in adolescence. To fill these research gaps, this study aims to prospectively examine (1) the development of SR and (2) their influence on adolescent-specific developmental outcomes in a large community sample.

**Methods/design:**

Based on previously collected data from the Potsdam Intrapersonal Developmental Risk (PIER) study with three measurement points, the present prospective, longitudinal study aims to add a fourth measurement point (PIER_YOUTH_). We aim to retain at least 1074 participants now between 16 and 23 years of the initially 1657 participants (6–11 years of age at the first measurement point in 2012/2013; 52.2% female). The study will continue to follow a multi-method (questionnaires, physiological assessments, performance-based computer tasks), multi-facet (assessing various domains of SR), and multi-rater (self-, parent-, and teacher-report) approach. In addition, a broad range of adolescent-specific developmental outcomes is considered. In doing so, we will cover the development of SR and relevant outcomes over the period of 10 years. In addition, we intend to conduct a fifth measurement point (given prolonged funding) to investigate development up to young adulthood.

**Discussion:**

With its broad and multimethodological approach, PIER_YOUTH_ aims to contribute to a deeper understanding of the development and role of various SR sub-facets from middle childhood to adolescence. The large sample size and low drop-out rates in the first three measurements points form a sound database for our present prospective research.

*Trial registration* German Clinical Trials Register, registration number DRKS00030847.

## Background

**Fig. 1:**
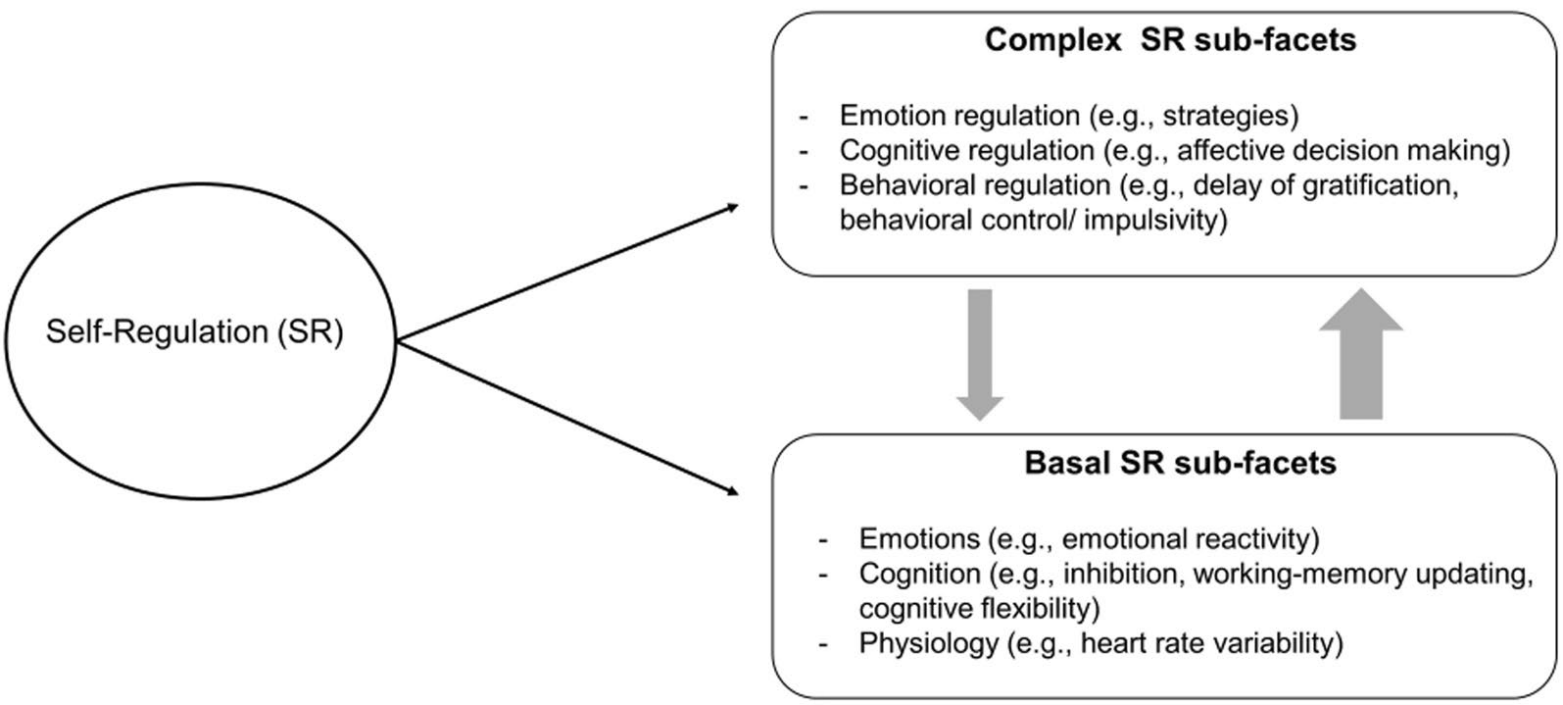
The conceptual hierarchical model of SR. The arrows indicate that a reciprocal relationship between basal and complex SR sub-facets is assumed. The line width indicates that the influence of basal on complex sub-facets is assumed to be stronger than vice versa

Self-regulation (SR) is defined as "…intrinsic processes aimed at adjusting mental and physiological state adaptively to context. [It] encompasses cognitive control, emotion regulation, and top-down and bottom-up processes that alter emotion, behavior, or cognition to attempt to enhance adaptation (or to achieve an explicit or implicit goal or goal state)” (p. 364) [1]. Various constructs are subsumed under this umbrella term [2]. These include basal physiological functions, such as the ability to adaptively regulate physiological responses captured via heart-rate variability (HRV), executive functions (EF) as basal (neuro-)cognitive SR sub-facets, as well as complex, potentially intentional processes, such as regulating one's own behavior or emotions [1, 3]. So far, there is no unifying theory of SR, which impedes scientific progress in this field [4]. In the majority of the proposed conceptual and taxonomic classifications, a hierarchical structure of the SR skills is assumed, with EF viewed as precursors of or basis for more complex SR skills such as planning [1, 4–6]. Thus, based on the theoretical assumptions of [1], and acknowledging that SR is a broad concept that encompasses various and also differently complex skills, we divided SR into more basal and more complex SR sub-facets that can be assigned to the regulation of emotion, cognition, behavior, and physiology. In our heuristic conceptualization of SR, we postulate that inhibition or heart rate variability belong to the basal SR sub-facets, whereas we consider delay of gratification or decision making as complex sub-facets. Basal and complex SR sub-facets are conceptualized as forming hierarchical but bidirectional relations (as indicated by the arrows in Fig. 1), and we assume that the influence of basal on complex sub-facets is stronger than vice versa.

Commencing in early childhood, SR skills dynamically develop during different stages of life at varying paces. Adolescence (i.e., from puberty till the early twenties; [7]) is a period of extensive changes in the expression, differentiation, and interconnectedness of the various SR competencies [8–11]. The development of SR from childhood to adolescence shows continuities and discontinuities, and little is known about their causes and predictors to date [12]. Furthermore, individual differences and external influences characterize SR competencies in adolescence [1, 10, 13]. SR is not only shaped by the individuals themselves but also by their social environment (i.e., parents and peers; [14–16]. In addition, the SR of an individual also affects the social environment [15], with these transactional person-environment relationships further driving individual development [17, 18].

There is consensus that the degree, differentiation, and interconnectedness of SR skills continue to unfold until young adulthood [8, 9, 11]. Based on our conceptual model, we postulate that during adolescence, complex SR sub-facets successively develop and unfold their potential [3, 10, 19]. This formation process of complex SR essentially builds on the expression of the basal sub-facets. Regulation processes are a fundamental driver of transactional person-environment relations that excel individual development [17, 18]. In adolescence, individual differences in SR become increasingly important as adolescents can choose their environment more independently. Thus, environment and behavior have a reciprocal relationship [18]. Emerging complex SR skills enable adolescents to identify appropriate goals, use planning strategies to achieve goals, and weigh short-term benefits against long-term consequences [4, 20]. Particularly in this age range, however, longitudinal evidence on the typical development of various SR sub-facets and its importance for the accomplishment of adolescent-specific developmental tasks is still scarce [12, 15, 21–24]. Our longitudinal research will therefore increase our understanding of the development and impact of SR, especially in the previously neglected but vulnerable period from puberty to early adulthood [25].

Research has consistently indicated positive associations between SR and various positive developmental outcomes, such as physical and mental health, well-being, or career success [26–32]. Consequently, a broad set of well-developed SR skills has been considered critical for adaptive development [33]. Similarly, low SR skills have been discussed as a "generic risk profile" [34–36]. That is, at least a certain level of SR skills is considered as a prerequisite of mastering relevant developmental tasks as well as everyday challenges. Adolescence is particularly important because it sets the course for adulthood by progress in social (e.g., the detachment from the parental home, establishment of romantic relationships), personal (e.g., the establishment of a stable identity), and achievement-related (e.g., school graduation, career choice) development [37]. Moreover, in this age range, challenges for SR arise from increasing personal responsibility as well as from the need to cope adequately with developmental tasks. Thus, adolescence is considered a potential turning point for adaptive or maladaptive development [38]: Adverse behaviors occur more frequently but develop very differently with remissions, aggravations, or chronifications (e.g., increased incidence of mental health problems; [39]). Additionally, some adolescents face a high comorbidity of mental health problems and an accumulation of developmental risks [40, 41].

However, a comprehensive meta-analysis on the role of SR in childhood and adolescence [30] has revealed that there is still a paucity of research that considered adolescent samples, as the majority of studies focused on early and middle childhood. Complicating matters further, researchers used different approaches to assess SR (e.g., parent and teacher reports; performance-based assessments), and the type of assessment was the most important moderator of the pooled effect sizes. In addition, many relevant outcomes that are highly relevant in adolescence, such as prosocial behavior, identity development, disordered eating behavior or experience of stigmatization, have not been adequately addressed. Because analyses have often followed an outcome-specific approach (i.e., focusing on single areas, such as aggressive or anxious behavior), little is known about the generic or domain-specific contribution of SR across different developmental outcomes. In addition, the opposite is also true: because analyses have often considered only one or a few sub-facets of SR, little is known about particular powerful sub-facets of SR and their interplay in predicting single or even multiple outcomes. Besides addressing the potential influence of (single and multiple) SR skills on various developmental outcomes, it is also relevant to explore which intra- and interpersonal factors (e.g., influence from significant others) can contribute to the individual level of SR in adolescence. Therefore, a comprehensive approach that concurrently includes multiple SR sub-facets and multiple outcomes over time is needed to analyze the dynamic and bi-directional association of SR with developmental outcomes in adolescence.

We will address these research gaps in the continuation of our prospective PIER-study (“Potsdam Intrapersonal Developmental Risk Study”) that has already captured the development from 6 to 13 years through three measurement points and will be extended to include the critical developmental period of late adolescence (16–21 years). Adding further measurement points will contribute to a comprehensive understanding of SR development from childhood to adolescence and its meaning for other areas of development.

## Methods/design

The PIER-study and its continuation as PIER_YOUTH_ is a prospective longitudinal study. Based on the previously collected data on participants in the PIER study (with three measurement points), this study extends collected data by a fourth measurement point which offers new developmental perspectives. To ensure longitudinal comparability while at the same time considering normative developmental changes, the previously applied methods/measures will be continued wherever possible and, if necessary, adjusted to the age of the participants (e.g., self-instead of parent-report). Furthermore, additional measures will be implemented to address additional central aspects of SR that become relevant in adolescence and young adulthood (e.g., planning behavior or risk taking). In short, PIER_YOUTH_ is a prospective, multi-method (encompassing physiological measures, performance-based computer tasks, questionnaires) multi-facet (including measures that assess cognitive, emotional, physiological and behavioral sub-facets of SR) and multi-rater (self- and parent report) approach to study the development and relevance of SR from middle childhood to late adolescence.

### Aims of the study

To close the aforementioned research gaps with respect to (1) the developmental course of SR and (2) the contribution of SR to different developmental outcomes in adolescence, PIER_YOUTH_ will address the following research questions:

- **Q1: Developmental trajectories of SR sub-facets.**
- **Q1.1:** *Which continuities and discontinuities emerge for the developmental trajectories of SR skills from middle childhood to adolescence?*
- **Q1.2:** *How can specific developmental patterns of SR be predicted?*
- ***Q1.3:**** How do individual SR skills develop in interaction with the level of SR skills in the peer group?*
- **Q2: Influence of SR skills on adolescence-specific developmental aspects.**
- ***Q2.1:**** How do SR skills in middle childhood affect relevant outcome variables in adolescence?*
- ***Q2.2:**** What is the contribution of SR combined with predictors previously established in literature?*
- ***Q2.3:**** What is the impact of SR in the context of peer groups (as a social norm) on outcomes?*

**Fig. 2:**
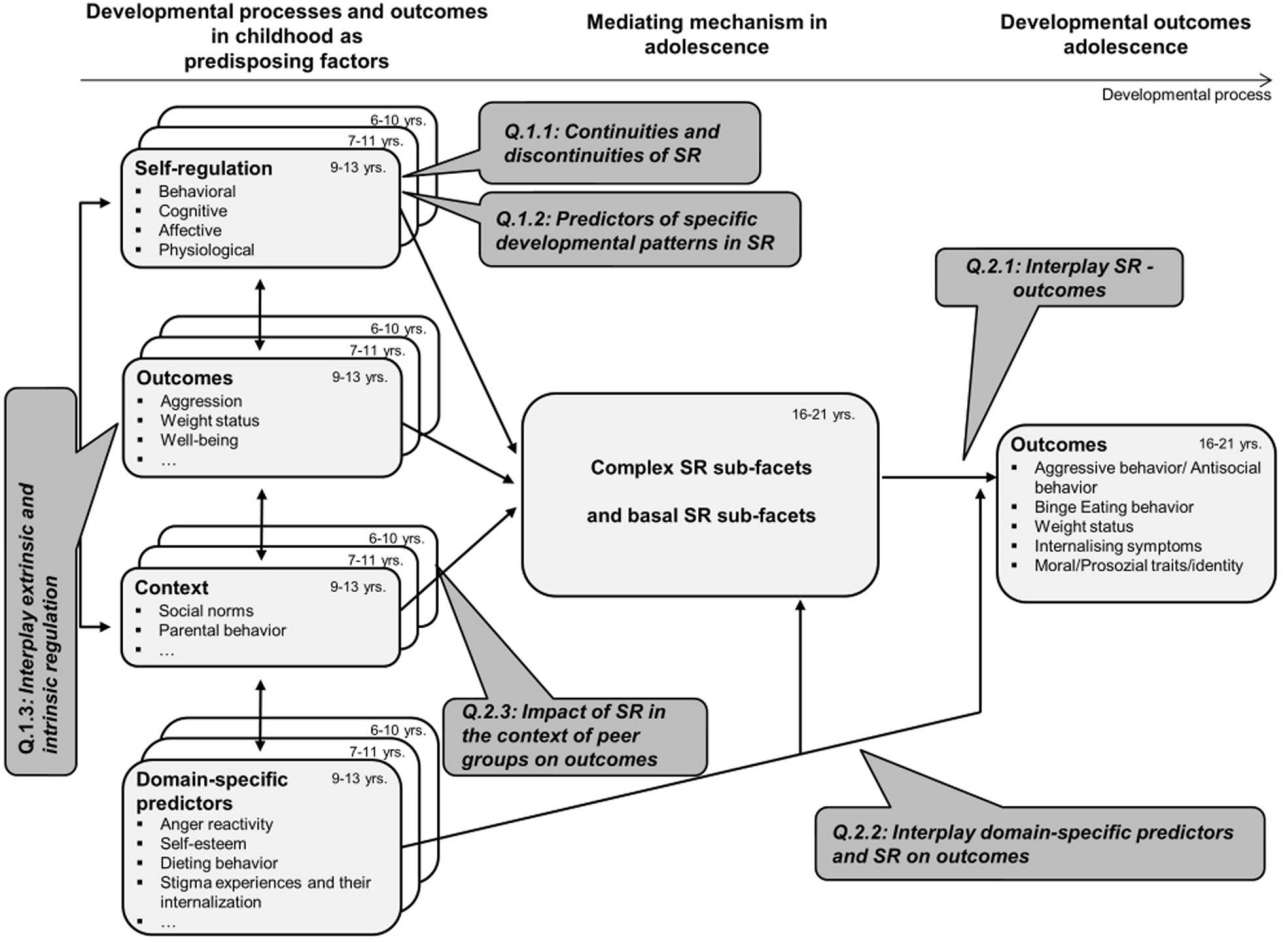
Exemplary overview of constructs collected longitudinally and research questions

We postulate that developmental processes and outcomes in middle childhood influence SR skills [Q1], which in turn influence outcomes in adolescence [Q1.2; Q2]. Furthermore, we assume bidirectional relations between contextual factors and SR [Q1.3]. With respect to Q2, we hypothesize that SR proves to be a central predictor for relevant developmental outcomes in adolescence. In terms of a cascade model, developmental outcomes can themselves become predictors, and diverse interactions between the predisposing factors in middle childhood are assumed. Figure 2 illustrates the previous and to-be-generated prospective database that can be drawn upon to answer these research questions in PIER_youth_.

### Study participants

The sample is based on the previously conducted PIER study with data from 1657 children (52.1% girls, average age: 8.36, *SD* = 0.95) at the first measurement point (T1) in 2011/2012. The aim was to recruit entire classes of elementary schools, if possible, to ensure economic data collection (several individuals tested at one location) in comparable social environments. The children were recruited in 120 classes (first to third grade) spread across 33 schools in the federal state of Brandenburg. To obtain a most representative sample, schools located in rural and urban areas and in regions with varying social stratification were included. This sample has been successfully followed-up over three years with only low drop-out rates at the second (T2) and third measurement points (T3; see Table 1). With respect to the forthcoming fourth PIER_YOUTH_ measurement point (T4), it can be assumed that due to the long interval since T3 (8 years) and the probable changes in school and residence that have occurred in the meantime, the drop-out rate will rise to a conservative estimate of around 30%. Therefore, we assume that 1074 adolescents and 750 parents will remain in the sample. Because not all participants aren’t still in school, no further data from teachers are collect at T4. Dependent on further funding, a fifth measurement point is planned three years later, aiming for 1020 participants.

**Table 1:** Overview of measurement points and available and future data

	PIER-study database (already collected)			PIER_YOUTH_
	T1 10/2011–03/2012	T2 10/2012–03/2013	T3 10/2014–03/2015	T4^a^ 08/2022–08/2023
*N* (children)	1657	1612	1534	1074
Drop-out rate (from the previous assessment point)	–	3%	5%	30%
Age (*M* ± SD; Range) in years	8.36 ± 0.95; 6–10 years	9.11 ± 0.93; 7–11 years	11.06 ± 0.92; 9–13 years	17.91 ± 1; 16–21 years
% female	52%	52%	52%	52%
*N* (parents)	1340	1197	1070	750
*N* (teachers)	1424	1175	1113	will no longer be assessed

^a^07/2022 Start of PIER_YOUTH_. Estimated values

### Recruitment strategy

We are contacting all eligible 1590 families (70 families provided at T3 no informed consent for further contact) from the PIER-study via mail to request their renewed participation. Due to the fact, that many former participants will be of legal age and due to the new General Data Protection Regulation (GDPR), it is necessary to obtain renewed written consent. Because the participants were minors at the former measurement points, we only have the address data of the parents. However, the majority of children have presumably not yet finished school or are still in contact with their parents. Therefore, it seems likely that they continue to live at home or can be contacted through their parents. If no valid contact data is available, the residents’ registration office will be contacted to disclose the latest address.

**Fig. 3:**
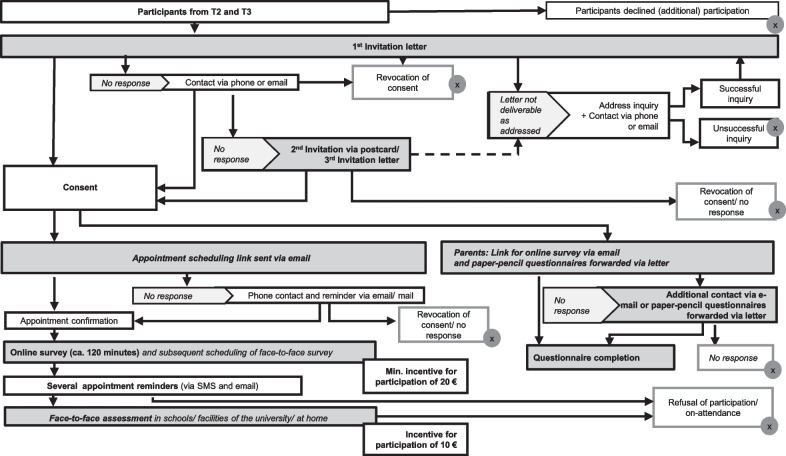
Overview of recruitment strategy. The symbol with the cross mark represents the exclusion from participation

Figure 3 shows an overview of the recruitment strategy. In order to obtain renewed informed consent, a letter including relevant study information and declaration of consent forms (in case of minors, also a parental consent form for their child’s participation) will be sent to the families. In case of no response within two weeks, we will re-contact the families by mail or telephone (if parents’ email addresses and telephone numbers are available). Particularly phone calls are an important means to get in direct contact with the families that also facilitates discussing and addressing potential barriers to renewed participation. Telephone calls will follow the spirit of motivational interviewing that has proven particularly helpful to ensure retention [42]. In case of unavailable mail/phone data or further lack of response after three weeks, the families will receive a reminder in the form of a postcard and, in case of further lack of response, a second invitation letter. We aim to contact all participants at least three times in order to realize a high retention rate. After providing written informed consent, participants will receive a link to arrange an online appointment for testing.

### Data collection strategy

In contrast to the initial plan of conducting one longer face-to-face session, data collection had to be adjusted due to contact restrictions following the Covid-19 pandemic. It is now taking place in two separate sessions, a longer online session followed by a short face-to-face onsite session. This procedure deviates from the prior data assessment at T1–T3 that took place face-to-face and in schools (two sessions per person, with a duration of around 50 min each). In PIER_YOUTH_, the young people will arrange an appointment for an online session with a trained test administrator. This online session will take about 2 h. At the beginning of the online session, the test administrator briefly explains the test instructions. Throughout the session, they are available for questions and activate the experiments (randomized in four blocks). The participants can complete the individual questionnaires and performance-based computer tasks at their own pace. The test administrator can see the participant’s progress, but not their answers or performance. At the end of the online survey, a 30 min face-to-face on site appointment will be arranged. During this session, the procedures that cannot be conducted online (e.g., objective measures, such as heart-rate variability (HRV), anthropomorphic measurements) will be carried out. As compensation, participants will receive a total of 30€. In addition, participants can win up to additional 15€ as part of two experiments (Balloon Analogue Risk Task (BART); distributive behavior task). To increase adherence to the face-to-face on site session, additional vouchers were raffled off for participation. If interested, participants will receive aggregated study results provided after data analysis. Ethical approval was obtained from the institutional review board of the University of Potsdam (8/84^th^ meeting—30.03.2020).

### Study measures

The PIER-study already included multiple measures of SR as self-reports, parent- and teacher-reports, and performance-based computer tasks. On a construct level, these assessments and objective measures continue in PIER_YOUTH_, although sometimes the specific assessment procedure had to be adjusted to the older age of the participants. In the following, not only the assessments at T4 will be described, but also the corresponding assessments at T1–T3 in order to clarify necessary age-dependent adjustments. An overview of the study measures is presented in Table 2. Of note, the questionnaires and performance-based tasks are presented to the participants in a block-randomized sequence in order to control for order effects (4 blocks). The computer tasks were programmed in E-Prime 2.0 Professional (2012 Psychology Software Tools) at T1–T3 and in JATOS/OpenSesame at T4. Online questionnaires were programmed with SoSci Survey (version 3.4).

**Table 2:** Overview of the measurement instruments across the measurement time points as well as reporting source

Construct	Measurement	Measurement time			
		T1	T2	T3	T4
*Basal self-regulation sub-facets*					
Inhibition	Fruit-Stroop task	PB	PB	PB	
	Stroop task				PB
	Control variables for Stoop task: items about color weakness/blindness				S
	Subscale inhibitory control of the temperament in middle childhood questionnaire (TMCQ)	P	P	P	
Working-memory updating	Digit Span Backward (ZN-R) subtest of German Wechsler Intelligence Scale for Children—Fourth Edition (WISC-IV)/Wechsler Adult Intelligence Scale—Fourth Edition (WAIS-IV) [age-appropriate version]	PB	PB	PB	PB
Cognitive flexibility	Cognitive flexibility task	PB	PB		
	Dimensional change card sorting (DCCS) [age-appropriate version]			PB	PB
Heart rate variability (HRV)	Polar watches (RS800CX, Polar Electro Oy, Kempele)	O	O		
	BioSign HRV-scanner				O
	Control variables for HRV: Questions about variables that might affect HRV-measurement: consume of caffeine/cardioactive substances; in-tensive physical training during the last 24 h; sleep quality and duration of the previous night; time of the last meal				S
Emotional reactivity	Subscale emotional control of the behavior rating inventory of executive function (BRIEF)	P	P	P	S, P
	Subscale anger/frustration of the temperament in middle childhood questionnaire (TMCQ)	P	P		
	Subscale anger of the Buss-Perry-aggression questionnaire				S
*Complex self-regulation sub-facets*					
Emotion regulation strategies	Subscale dealing with anger of the questionnaire to elicit emotion regulation in children and adolescents (FEEL-KJ)	S, P	S, P	S, P	
	Subscale emotion regulation with anger of the intelligence and development scales (IDS)	S	S		
	Emotion regulation questionnaire (ERQ)				S
	Cognitive emotion regulation questionnaire (CERQ)				S
Affective decision-making	Hungry donkey task [age-appropriate version]	PB	PB	PB	PB
Delay of gratification	Decision questions with real incentives (sweets and toys)	PB	PB	PB	
	German delay discounting test (DDT)				S
Behavioral control/impulsivity	UPPS impulsive behavior scale (short-version)				S, P
	Self-regulation scale (SRS)				S, P
	Balloon analogue risk task (BART)				PB
Planning behavior	Subscale plan/organize of the behavior rating inventory of executive function (BRIEF)	T	T	T	S, P
	Subscale impulsivity of the barratt impulsiveness scale (BIS)				S
	UPPS impulsive behavior scale (short-version)				S, P
Conscientiousness	Subscale conscientiousness of the big five inventory-SOEP (BFI-S)				S
Eating-related SR	Children's/adult eating behavior questionnaire (CEBQ/AEBQ)	P	P	P	P, S
	Subscale external eating behavior of the Dutch eating behavior questionnaire (DEBQ)	P	P	P	S, P
	Subscale restrictive eating behavior of the Dutch eating behavior questionnaire (DEBQ)	S	S	S	S
	Determination of losing weight and changing eating behaviors				S
	Subscale emotional eating of the eating behavior disturbance questionnaire			S	
*Outcome measures and predictors*					
Weight status	Weight and height	P, O	P, O	P, O	S, O
Body dissatisfaction	Body silhouettes [age-und gender-appropriate version]	S	S	S	S
	Male muscularity silhouettes [for males only]				S
	Subscale attitudes of the drive for muscularity scale (DMS)				S
	Current estimation of one’s own weight status				S
	Weight satisfaction	S	S	S	S
Internalized weight stigma	Weight bias internalization scale (WBIS-C)	S	S	S	S
Stigma	Perception of teasing scale (POTS)	S^a^	S^a^	S	S
	Appearance-related social pressure questionnaire (FASD)				S
Self-esteem	Subscale self-esteem of the questionnaire of assessing health-related quality of life (KINDL)	S	S		
	Subscale self-esteem of the child health questionnaire (CHQ)			S	
	Rosenberg’s self-esteem scale				S
Prosocial Behavior	experiment on distributive behavior				PB
	Questionnaire of prosocial behavior				S
	Subscale prosocial behavior of the strengths and difficulties questionnaire (SDQ)	P, T	P, T	P, T	S, P
Moral identity	Questionnaire for moral identity				S
Justice sensitivity	Justice sensitivity inventory for children and adolescents (JSI-CA 5)				S
Loss of Control Eating and Binge Eating	SCOFF	S/P^b^	S/P^b^	P, S^c^	S, P
	Questionnaire of eating and weight patterns (QEWP)	S	S	S	S
	binge eating and compensatory behaviors as key behavioral features of eating disorders via eating disorder examination (EDE-Q)				S
	Loss of control over eating scale (LOCES)				S
Emotional and behavioral problems	Strengths and difficulties questionnaire (SDQ)	P, T	P, T	P, T	S, P
Depressive symptoms	Diagnostic classification systems for mental disorders in childhood and adolescence of the ICD-10 and DSM-IV (DISYPS-KJ)	S	S		
	Depression test for children of elementary school age (DTGA)			S	
	Patient health questionnaire (PHQ-8)				S
Anxiety symptoms	Mannheim adolescent questionnaire (MJF)	S	S		
	Child anxiety test II (KAT-II)			S	
	Generalized anxiety disorder screener (GAD-7)				S
Social information processing	Scenes for social information processing in adolescence (SSIPA)				S
Aggressive and antisocial behavior	Adult/youth self-report (ASR/YSR), youth self-report; adult/child behavior checklist (ABCL/CBCL)	P	P	P	S, P
	Children’s social behavior scale (CSS-T)	T	T, S	T, S	S, P
	Instrument for reactive und proactive aggression (IRPA)	T	T	T	S, P
*Other variables*					
Sociodemographic variables	Age, gender	S, P	S, P	S, P	S, P
Socioeconomic status and education	Educational attainment of mother and father	P^e^	P	P	
	Occupation of both parents, household size, monthly household income			P	
	Winkler-index				P
	Subjective perceived social status via McArthur scale				S
Contact with parents	Living space of the child contact frequency with their child				P
Migration background	Language spoken with child/at home; participants’ and parents’ country of birth	P	P	P	S
Pubertal status	Items about sex-specific bodily changes during puberty				S
Cognitive theory of mind	Cartoon task	PB	PB		
	Belief-desire reasoning task			PB	PB
Affective theory of mind	Cartoon task	PB	PB		
	Faces scale of Cambridge mindreading face-voice battery			PB	PB
Cognitive processing speed	Digit symbol coding test of the German Wechsler intelligence scale for children—fourth edition (WISC-IV)/Wechsler Adult Intelligence Scale (WAIS-IV) [age-appropriate version]	PB	PB	PB	PB
Family risk factors	Family adversity index (FAI)		P	P	P
Current burden	Questions about perceived extent of corona-related stress as well as the stress caused by the current global political situation				S
Use of mental health services	Questions about adolescents’ utilization of therapy				S, P
Level of personal functioning	Operationalized psychodynamic diagnostics—structural questionnaire short version (OPD-SQS)^d^				S
Social media use	Questions about time spent on physical activity and media	S, P	S, P	S, P	S, P
	Adolescents’ usage of social media (frequency and duration of series consumption; active and passive social media use)				S
	Subscale media pressure from the sociocultural attitudes towards appearance questionnaire IV-R (SATAQ IV-R)				S
Academic performance	Potsdam Teacher Questionnaire (school skills of the child)	T	T	T	
	Grades in reading and spelling (T1–T3) and mathematics (T3)	P	P	P	
	Perceived academic accomplishments relative to peers via visual analog scale				S
Social desirability	Short version of the scale for detecting test manipulation through faking good and social desirability bias (SEA-K)				S
Parental weight status	Parent report about their own body weight	P	P	P	

^a^Short version, performance related teasing excluded; ^b^combination of self- and parent-report; ^c^only three out of five items; ^d^provided in a subsample of participants (additional link provided at the end of the online-session for independent and voluntary completion; ^e^net household income excluded; *S* Self-report, *PB* Performance-based computer task, *O* Objective measures, *T* Teacher-report, *P* Parent-report

### Self-regulation

As mentioned above, SR skills were divided in basal and complex SR sub-facets.

#### Basal SR sub-facets

Basal SR sub-facets include inhibition, working-memory updating, cognitive flexibility, heart rate variability (HRV), and emotional reactivity. Inhibition, cognitive flexibility and emotional reactivity are collected online, while the HRV and working-memory updating are collected in the face-to-face on site assessment.

##### Inhibition

The ability to control, modulate, and plan one’s behavior, to suppress primary behavioral impulses and inappropriate responses, and to resist impulsive behavior was assessed at T1–T3 via parent-ratings with six items from the Inhibitory Control subscale of the Temperament in Middle Childhood Questionnaire [43]. The ability to suppress primary behavioral impulses in favor of a less dominant response was also assessed with a performance-based measure at T1–T3 using an adaptation of the Fruit-Stroop Task [44, 45]. At T4, an age-appropriate computer-based Stroop Task is used [46]. Against a black background, participants see a color word (RED, BLUE, GREEN) printed in different colors (red, blue, green). After the color word appears, the participants have to name the ink-color as quickly as possible. The first 42 trials are performed without response conflicts (words replaced by rows of three to five asterisks), followed by runs with color words. The initial 42 color words are presented with congruent ink, and the following 42 words with incongruent ink, which requires the inhibition of a predominant response (e.g., reading the color word) to correctly name the ink-color. Inhibition capacities are measured via interference scores calculated from reaction times or accuracy in congruent relative to incongruent trials. Preceding the Stroop test at T4, potential color-vision deficiency is assessed via self-report with two self-constructed items: whether the adolescent is struggling with identifying colors and, if applicable, from which color-vision deficiency they are suffering.

##### Working-memory updating

At T1–T3, the ability to actively monitor and manipulate information in short-term memory was assessed using the Digit Span Backward (ZN-R) subtest of the German version of the Wechsler Intelligence Scale for Children—Fourth Edition (WISC-IV; 6–16 years; [47]). At T4, the corresponding subtest of the Wechsler Adult Intelligence scale—Fourth Edition (WAIS-IV; from 16 years; [48]) as an age-appropriate version is used. The participants hear a series of individual spoken digits and are asked to verbally repeat the digits in reverse order. After a correct repetition, the following series is extended by one digit, and after two incorrect turns, the test is terminated. The measure for updating capacity is the length of the final sequence or the number of sequences repeated correctly in reversed order.

##### Cognitive flexibility

The ability to adjust the attentional focus to current demands or to flexibly shift between mental sets and demands was assessed via the child-appropriate, performance-based Cognitive Flexibility Task [49] at T1 and T2. From T3 onwards, the age-appropriate performance-based Dimensional Change Card Sorting (DCCS) task is being used [50]: On a computer screen, participants are presented with two geometrical symbols that vary across the two dimensions ‘shape’ and ‘color’ (i.e., a blue box and yellow star). A third symbol with complimentary features (i.e., blue star) must then be matched as quickly as possible to one of the two original shapes via keypress according to a fixed rule indicted by a verbal cue (i.e., ‘shape’ or ‘color’). The 20 trials of the first block establish a dominant rule (i.e., 50% of participants sort by shape or color, respectively). In the second block, trials appear in pseudo-random order: 45 non-switch trials follow the dominant rule and 15 switch trials require the other, non-dominant rule. There is always at least one dominant trial between two non-dominant trials. The costs of switching to the new sorting rule are calculated from reaction times or accuracy in the switch relative to the non-switch trials. High cognitive flexibility comes with low switch costs.

##### Heart rate variability (HRV)

HRV, as a measure of neurovegetative activity and autonomic function of the heart, characterizes variations in heart rate over a given measurement period. High HRV is considered a biological marker of good self-regulatory capacity [51, 52]. HRV was collected at T1 and T2 using electrodes on the palms of the hands for a period of 3 min using Polar watches (RS800CX, Polar Electro Oy, Kempele) [53]. From T4 onwards, the heart rate is being objectively measured using clamp electrodes applied on the participants’ wrists and the software BioSign HRV-Scanner standard professional version 3.5 [54] for a period of 5 min. Furthermore, a number of external factors that may influence HRV are assessed as self-report. With self-constructed items, adolescents indicate whether they have (1) consumed caffeine in the previous 2 h, (2) consumed other cardioactive substances (such as nicotine, alcohol, other drugs) or undergone intensive physical training during the last 24 h. Moreover, three open questions are used to assess the quality and duration of sleep during the previous night, and the time of the last meal.

##### Emotional reactivity

The tendency to show strong emotional reactions in general was recorded at T1–T3 with the subscale *emotional control* of the Behavior Rating Inventory of Executive Function (BRIEF; [55]) via parent-report. From T4 onwards, the same scale is used to assess emotional reactivity via self- and parent-report. The proneness to react with anger in the face of interruption of tasks or hindrance in goal achievement in particular was recorded at T1 and T2 using seven items of the Anger/Frustration subscale of the Temperament in Middle Childhood Questionnaire (TMCQ; [43]) via parent-report. From T4 onwards, anger reactivity is assessed as self-report via six items (as suggested by [56]) of the *Anger* subscale of the Buss-Perry Aggression Questionnaire (German: [56]).

#### Complex SR sub-facets

Complex SR sub-facets include emotion regulation strategies, affective decision making, delay of gratification, risk-taking/impulsivity, planning behavior, conscientiousness, and eating-related SR. All these assessments are conducted during the online session.

##### Emotion regulation strategies

At T1–T3, anger-regulation strategies (dispersion, perseveration, expression, control, external regulation) were recorded via age-appropriately adapted items of the Dealing with Anger subscale of the Questionnaire to Elicit Emotion Regulation in Children and Adolescents (FEEL-KJ; [57]) via self- and parent-reports. Furthermore, at T1 and T2 strategies of dealing with anger were assessed via an adaption of the Emotion Regulation subscale of the Intelligence and Development Scales (IDS; [58]) via self-reports. At T4, the German versions of the Emotion Regulation Questionnaire (ERQ; [59]) and the 27-item version of the Cognitive Emotion Regulation Questionnaire (CERQ; [60]) are used to capture self-reported emotion-regulation strategies, namely cognitive reappraisal and expressive suppression (ERQ) as well as “functional” (e.g., relativizing, refocusing) and “dysfunctional” (e.g., self-blame, rumination) strategies (CERQ).

##### Affective decision-making

The tendency to make emotion-driven decisions and take risks was assessed at T1–T3 using the computer-based Hungry Donkey Task [61]. At T4, we use an age-appropriate version with a higher number of trials and a more complex pattern of win-loss probabilities [62]. Participants are asked to help a hungry donkey and to collect as many apples as possible by selecting and opening one of four depicted doors in 100 trials. After opening a door, certain numbers of gained (green) and lost (red) apples are displayed (e.g., + 4, − 8). The exact win-loss probability is different for each door and unknown to the participants. Relevant measures are the number of apples obtained (net gain) or the difference of choices for “disadvantageous” door positions (yielding higher immediate gains but long-term losses) versus “advantageous” door positions (yielding lower immediate gains but long-term gains) across trials (e.g., all trials or in learning blocks with several trials each). A preference for disadvantageous doors reflects poorer affective decision making, a preference for immediate rewards, or a stronger insensitivity to negative consequences.

##### Delay of gratification

The ability to forgo an immediate reward and to control impulses in favor of a larger or higher value in the future was assessed at T1–T3 using four decision questions [63]. Children needed to decide whether they wanted to obtain a smaller amount of real incentives (2 sweets, 2 toys) immediately or a larger amount some time later. At T4, the German Delay Discounting Test (DDT; [64, 65]) is used. In 27 items, participants are asked to imagine they would receive money from the test administrator, and they must choose between a smaller, immediate amount of money or a larger amount of money to be received later. The derived discount rate informs on how much a person devalues a future reward (referred to as delay discounting). The discount rate depends, among other variables, on the value of the immediately available reward and the delay interval. A low devaluation of future rewards speaks for higher delay-of-gratification ability.

##### Behavioral control/impulsivity

At T4, this facet is assessed via self- and parent-reports, using a short version of the UPPS Impulsive Behavior Scale (UPPS; adapted from [66] and [67]; 10 items on four subscales: lack of premediation, urgency, sensation seeking, and (lack of) perseverance) and the Self-Regulation Scale (SRS, 10 items; [68]). The SRS captures the ability to sustain difficult actions even when attention or motivation is impaired. Furthermore, at T4, risk-taking is assessed using the computer-based Balloon Analogue Risk Task [69] as a behavioral measure with 20 trials. The task consists of inflating a balloon displayed on a computer screen by pressing a button repeatedly, with each press yielding a small monetary reward (0.5 Cent) that the participants actually receive after the test session. After each key press, participants have to decide either to continue inflating the balloon and thus increase their monetary reward, or to stop inflating it. With further inflation, participants increase the risk that the balloon will burst (in which case all money of this trial is lost), whereas termination secures the money earned in this trial. As dependent variable the so-called adjusted average pumps, i.e., pumps on the balloons which did not explode, will be calculated.

##### Planning behavior

At T1–T3, the child’s ability to deal with tasks that have to be mastered now or in the future was measured via teacher-reports with the Plan/Organize subscale of the BRIEF [55]. At T4, this subscale is included as a self- and parent-report. Additionally, planning behavior is assessed with the two Intention-items of the UPPS (adapted from [66, 67]) described above. Furthermore, five items of the Impulsivity subscale from the German version of the Barratt Impulsiveness Scale (BIS) (German: [70]) are assessed via self-report.

##### Conscientiousness

Conscientiousness, one of the personality dimensions of the Big Five, is considered as self-regulatory competence in personality psychology [71]. Conscientiousness refers to the tendency to be disciplined, perform well, and be reliable. At T4, it is assessed using parsimonious subscale of a short version the Big Five Inventory-SOEP (BFI-S; [72]) via self-report (3 items).

##### Eating-related SR

SR competencies associated with eating behavior that are regarded as contributing to weight status were assessed at T1–T3. The emotional overeating, emotional undereating, food responsiveness, and satiety responsiveness subscales of the Children’s Eating Behavior Questionnaire were assessed via parent-report (CEBQ; [73]). Moreover, at T3, a short version of the restrictive eating behavior subscale of the Dutch Eating Behavior Questionnaire for children (DEBQ; [74]) and six items of the Eating Behavior Disturbance Questionnaire [75] assessing emotional eating were used (self-reports). Furthermore, the subscale external eating behavior of the DEBQ [75] was assessed via parent-report. At T4, the CEBQ is assessed via parent-report, and the German version of the Adult Eating Behavior Questionnaire (AEBQ; [76, 77]) is applied as self-report. An age-appropriate version of the DEBQ is used to assess restrictive eating behavior via self-report and external eating behavior via parent-report [78]. Furthermore, the survey is assessing food self-regulation via self-report. On two self-constructed slider scales, the participants are indicating their determination of losing weight and changing eating behaviors in the near future.

#### Outcome measures and predictors

Outcome measures and predictors encompass multiple measures that are of importance in adolescence, amongst others weight status, body dissatisfaction, stigma, prosocial behavior, moral identity, emotional and behavioral problems, as well as social information processing.

##### Weight status

Children’s weight status was measured at T1–T3 using parent-reports and objective anthropometric measurements of height and weight (dressed but without jacket/shoes). Body weight was assessed with the personal floor scale Kern MPB 300K100, and the Stadiometer seca 217 measured the participants’ body height. To ensure privacy, measurement and weighing were performed individually and, if necessary, behind a folding screen. At T4, this standardized protocol is implemented during the on-site session. Self-reported weight is additionally collected with one self-constructed item in the online survey.

##### Body dissatisfaction

Body dissatisfaction was assessed at T1–T3 using child-specific body silhouettes [79]: Children were asked to indicate their current and their desired body figure using seven sex-specific body silhouettes with ascending degrees of relative fat mass. The difference between the two selected silhouettes can be used as indicator of body dissatisfaction [80]. In order to account for physical changes during adolescence, nine sex-specific silhouettes of Thompson and Gray [81] are applied at T4. To also cover aspects relevant for body images among males, nine additional silhouettes from Lynch and Zellner [82] are applied to map muscularity. The attitude subscale of the German Drive for Muscularity Scale [83] is assessing the muscularity related dissatisfaction of male and female participants. In addition, at T1–T4 single items are assessing the participants’ weight satisfaction and relevance of weight and one self-constructed item rates the current estimation of one’s own weight status (reaching from “very underweight” to “very overweight) as self-report.

##### Internalized weight stigma

At T3, internalized weight stigma was assessed using the child-adapted version of the Weight Bias Internalization Scale (WBIS-C; [84]). This scale is also used atT4.

##### Stigma

At T1–T3, perceptions of being teased by others about one’s weight were assessed via self-report using an abbreviated version of the appearance-related subscale of the Perception of Teasing Scale (POTS; [85]). Additionally, it was assessed from which persons (groups) the stigmatizations originated. At T3–T4, weight-related and performance-related teasing is being measured via the POTS; with additional items assessing suffering caused by each teasing form (POTS; [85]). Furthermore, at T4 the two subscales exclusion and parental encouragement of the appearance-related social pressure questionnaire (FASD; [86]) are used with four items each.

##### Self-esteem

The positive view of oneself was recorded at T1 and T2 with the Self-Esteem subscale of the health-related quality of life questionnaire (KINDL; [87]) and at T3 with the Self-Esteem subscale of the Child Health Questionnaire (CHQ; [88, 89]). At T4, the three-item Rosenberg’s self-esteem scale [90] is used.

##### Prosocial behavior

At T1–T3, prosocial behavior was measured via parent- and teacher reports via the accordant subscale of the Strengths and Difficulties Questionnaire (SDQ) (five items; [91, 92]). At T4, it is continued as a self- and parent report. In addition, prosocial behavior will be additionally assessed in an age-appropriate experiment with a particular focus on distributive behavior. In line with previous research in adult samples [93, 94], participants are requested to distribute a constant amount of money (7 Euro) between themselves and other participants under three conditions (dictator game, ultimatum game, third-person punishment scenario). Participants receive the actual money that they have kept for themselves from one randomly selected condition together with the money for the study participation. Finally, we assess prosocial behavior via four items asking about the adolescents’ actual prosocial behavior in everyday situations [95].

##### Moral identity

Following procedures in adolescence [96] and adulthood [97] at T4, participants are being asked to rate the personal importance of 18 positive, partially morality-related traits (e.g., being honest, fair) to assess the relative importance of moral traits [98].

##### Justice sensitivity

At T4, sensitivity to injustice from the victim’s, observer’s, and perpetrator’s perspective will be assessed via self-reports with five congruently worded items per scale from the Justice Sensitivity Inventory for Children and Adolescents (JSI-CA 5; [99]).

##### Loss of control eating and binge eating (BE)

At T1–T3, the SCOFF (5 items; [100]), allowing for a categorical (conspicuous/inconspicuous) and a dimensional view of abnormal eating behavior, was used as a screening instrument to detect conspicuous eating behavior as self- and parent-report. For specific consideration of eating behaviors associated with Binge Eating (BE), an age-adapted version of the Questionnaire of Eating and Weight Patterns (QEWP; [101, 102]) was used. At T4, these survey instruments are continued as self-reports, together with the SCOFF as a parent version. Furthermore, binge eating and compensatory behaviors as key behavioral features of eating disorders are assessed via self-report with six items of the German version of the Eating Disorder Examination Questionnaire (EDE-Q; [103]). Based on these answers, branching items of the EDE-Q assess the frequency of binge eating and compensatory behaviors. The Loss of Control Over Eating scale (LOCES; [104, 105]) with seven items is also included.

##### Emotional and behavioral problems

At T1–T3, we used the Strengths and Difficulties Questionnaire (SDQ; [91, 92]) with the four subscales emotional problems, conduct problems, hyperactivity, and peer problems (parent- and teacher-report). Further, the SDQ encompasses the potential impairment caused by symptoms. At T4, the age-appropriate version SDQ-18+ is implemented as a self-report measure. In addition, the parent-report of the SDQ is also applied to allow for continuity in reporting, while also controlling for informant effects. The SDQ can be evaluated as a total problems scale and at subscale level.

##### Depressive symptoms

Using the diagnostic classification systems for mental disorders in childhood and adolescence of the ICD-10 and DSM-IV (DISYPS-KJ; [106]), children rated four dichotomous items (yes/no) on depressive symptoms at T1 and T2. At T3, depressiveness was assessed with six items of the Depression Test for Children of Elementary School Age (DTGA; [107]). From T4 onwards, the Patient Health Questionnaire, PHQ-8 (i.e., PHQ-9 without the item on suicidality; [108, 109] is used (self-report). The PHQ-8 includes eight items that map the diagnostic criteria of depression according to the DSM and thus the presence and severity of depressive symptoms [109].

##### Anxiety symptoms

At T1 and T2, anxiety was assessed using 11 items of the Mannheim Adolescent Questionnaire (MJF; [110]) and at T3 using six items of the Child Anxiety Test 2 (KAT-II; [111]). At T4, the Generalized Anxiety Disorder Screener (GAD-7; [112]), a seven-item instrument for assessing symptoms of generalized anxiety disorder according to the DSM, is used (self-report).

##### Social information processing (SIP)

At T4, individual specifics of the cognitive processing of social information (in different processing steps) are assessed with an adapted version of the Scenes for Social Information Processing in Adolescence (SSIPA; [113]) survey. Adolescents are given three age-appropriate descriptions of potentially provocative social situations, each followed by two questions indicating the participant’s interpretation of each situation (likelihood of hostility/neutral intention on the part of the protagonist), five items assessing triggered emotions (e.g., anger, sadness, embarrassment), their evaluation of four possible responses (assertiveness, passivity, overt/relational aggression) with five items each assessing likelihood of choosing each of the responses. From this, scores are calculated for specific processing styles for different SIP steps.

##### Aggressive and antisocial behavior

At T1–T3, antisocial behavior was assessed using the delinquent behavior subscale of the Child Behavior Checklist (CBCL/4–18; [114, 115]) via parent-report. At T4, parents rate their child’s antisocial behavior with 17 items from the Adult Behavior Checklist (ABCL; [116, 117]) and two items from the CBCL [114, 118]. Adolescents self-report on antisocial behavior with the items from the Adult Self-Report (ASR; [116, 117]) and one additional item from the Youth Self-Report (YSR; [114, 118]). Forms (physical, relational, verbal) and functions (proactive, reactive) of aggressive behavior were assessed at T1–T3 via teacher-reports and at T3 via self-reports, using seven and six items from the Children’s Social Behavior Scale (CSBS-T; [119]) and the Instrument for Reactive and Proactive Aggression (IRPA; [120]), respectively. At T4, both questionnaires on aggressive behavior are used as self- and parent-reports.

#### Other variables

In addition, a number of variables are assessed that may be included in the analyses as grouping or control variables.

##### Sociodemographic variables

Age and gender of the participants are recorded in the self- and parent-reports. At T4, the gender category “diverse” is added to “male” and “female”.

##### Socioeconomic status (SES) and education

At T1–T3, SES was estimated using the educational attainment of the mother and father, on a scale from 1 (missing middle school degree) to 6 (university degree). At T3, the occupation of both parents, the household size, and the monthly household income were additionally recorded. At T4, subjectively perceived social status is assessed as self-report with the McArthur Scale [121], because the subjective assessment of relative status has proven to be a particularly good indicator of social position. Adolescents are indicating on a “ladder” where they locate their social position relative to others. Furthermore, they report their current occupation (student, trainee, etc.). In addition, parents provide information on the Winkler-Index [122] which reflects the educational, occupational, and financial background of the families.

##### Contact with parents

At T4, parents are asked whether their child still lives with them (or already moved out) and how often they are in contact with their child.

##### Migration background

At T4, to determine migration background, the adolescents report about the language spoken at home, as well as their and their parents’ country of birth.

##### Pubertal status

At T4, two self-constructed items are used to assess sex-specific bodily changes during puberty. Adolescents rate the concurrency of their body changes in comparison to their peers on a 5-point Likert-scale ranging from significantly earlier to significantly later. Additionally, females indicate whether they have experienced their first menstruation, and males whether their voice has changed already.

##### Cognitive theory of mind

The ability to infer action intentions and beliefs of others was assessed at T1 and T2 with six items of a cartoon task [123]. At T3 and T4, the computer-based Belief-Desire Reasoning Task [124] is applied. In this task with 30 trials, participants receive information about another person’s preferences (e.g., she likes/does not like apples) and assumptions about the state of reality (e.g., she thinks that the apples are in the blue/yellow box) and are then asked to choose which of the two boxes that person will open.

##### Affective theory of mind

The ability to recognize the emotional states of others was assessed at T1 and T2 with six items of a cartoon task [123]. From T3 onwards, the German version of the computer-based Facial Scale of the Cambridge Mindreading Face-Voice Battery [125] is applied. Participants watch 5.5 s long, silent movie clips in which actors portray various complex emotions (e.g., pensive, confused). Subsequently, participants are asked to select (by pressing a key) one adjective that best describes the emotion from four adjectives presented on the screen. At T4, 14 of the videos show adult actors, and six videos show adolescent actors.

##### Cognitive processing speed

Due to strong overlaps with some cognitively anchored self-regulatory competencies, cognitive processing speed was measured using the Digit Symbol Coding Test (ZST) of the German version of the Wechsler Intelligence Scale for Children—Fourth Edition (WISC-IV; 6–16 years; [47]) at T1–T3. At T4, the corresponding subtest of the Wechsler Adult Intelligence scale—Fourth Edition (WAIS-IV; from 16 years; [48]) as an age-appropriate version is used. Participants are asked to assign abstract symbols to a series of numbers using a given coding key. The number of correct matches produced in a limited time (120 s) is measured.

##### Family risk factors

At T2 and T3, the Family Adversity Index (FAI; German adaptation after [126]) was used to record various critical life events of the child over the entire life span via parent reports with dichotomous items (*yes*/*no*) (e.g., including massive partnership conflicts of the parents, mental disorder of a parent). At T4, these questions are expanded by further critical life events, and parents are asked to rate them separately concerning different life phases of their child (until the 6th birthday and in in the last five years).

##### Current burdens

T4 takes place during the COVID-19 pandemic and the war in Ukraine, both of which are associated with increased psychosocial stress for children, adolescents and their families [127, 128]. Therefore, the perceived extent of corona-related stress as well as the stress caused by the current global political situation are assessed via self-report on a 7-point Likert-scale ranging from ‘not burdensome at all’ to ‘extremely burdensome’.

##### Use of mental health services

At T4, a self-constructed item explores adolescents’ utilization of therapy until T4 (self- and parent-report). If applicable, more specific information is requested (e.g., type of therapy, reasons for therapy, duration of therapy).

##### Level of personality functioning

At T4, the Operationalized Psychodynamic Diagnosis—Structure Questionnaire Short Version (OPD-SQS; [129]) assesses the level of personality functioning in terms of perception, control, communication and attachment (self-report).

##### Social media use

At T1–T4, parents and children/adolescents rate the child’s/adolescents’ time spent on average on physical activity, watching television, and using a computer (items adapted from the KiGGS-study (a cohort longitudinal study on the health of children, adolescents and young adults in Germany; [130, 131]). At T4, additional self-report items are assessing the adolescents’ usage of social media (frequency and duration of series consumption; active and passive social media use according to [132]; type of media used). Another two self-report items of the Sociocultural Attitudes Towards Appearance Questionnaire-IV (SATAQ‐IV-R [133]; German Version: [134]) are assessing media pressure experienced by the adolescents.

##### Academic achievement

At T1–T3, teachers rated a child’s abilities regarding grammar, concentration, memory, reading, spelling, numeracy and reasoning (on a scale of 1–6 corresponding to school grades) using the Potsdam Teacher Questionnaire [135]. Additionally, parents indicated their child’s grades in German reading and spelling (T1–T3) and mathematics (T3). At T4, adolescents indicate how they perceive their academic accomplishments relative to their peers based on a 10-point visual analog scale in the form of a hierarchical ladder (from 0 “on a par with people with the worst performances” to 10 “on a par with people with the best performance”).

##### Social desirability

At T4, the short version of the scale for Detecting Test Manipulation through Faking Good and Social Desirability Bias (SEA-K; [136]) is included in the self-report questionnaire.

##### Parental weight status

At T1–T3, parents were asked to report their height and weight to calculate their body mass index (BMI; [137]).

### Statistical analyses

Before testing specific hypotheses, several methodological challenges have to be considered. Firstly, this concerns the age-appropriate operationalization of constructs while maintaining conceptual equivalence (e.g., adolescence-specific content adjustments or change of measures; the transition from parent- to self-report). Secondly, the concepts itself can change (e.g., the dimensional structure of SR becomes more complex with increasing age). To deal with these problems, we use multiple statistical approaches like confirmatory factor analysis for measurement invariance testing, developmental scaling, approximate invariance testing, or directly including the change of structure in the model.

Thirdly, methodological challenges arise from the integration of multiple indicators of the same construct due to the comprehensive multi-method and multi-rater assessment of the SR skills. The comprehensive and concurrent examinations of the SR-sub-facets (including self-report, proxy-report (parents, teachers), and behavioral data) will form the basis of the data-driven synthesis of the different assessment methods [138]. This way, latent factors of individual SR competencies with ratings from different sources can be identified. In addition, whenever possible, we will model method factors in latent analyses to account for rater effects. Fourthly, the inclusion of several related constructs may lead to multicollinearity between SR-sub-facets. We will address this problem via various established approaches. One is exploratory factor analyses to analyze the empirical relations between variables and to reduce the number of variables (Eisenberg et al. [138]). As another approach, we will apply a data-driven focus after testing for multicollinearity, in case no theoretical or evidence-based assumptions integrating the different sub-facets can be derived from the literature. Additionally, effect sizes provide information on the strength of the relations, which facilitates interpreting the clinical significance of the findings. Furthermore, we will conduct subgroup analyses (e.g., age; gender; pubertal status, see [139]) to make statements about the generalizability of the findings. For further hypotheses testing, we will apply different structural equation models (e.g., cross-lagged analyses, latent-growth modelling). If possible, the analyses will be performed at the latent level. School class membership will be accounted for when necessary and possible. The calculations will be carried out with SPSS, R, and M*plus*.

#### Power analysis

Because the present study bases on existing prospective data, a power calculation can only examine whether the existing (*N* = 1657) and expected samples (*N* = 1074 for T4, *N* = 1020 for T5) are sufficient for the intended analyses. A Monte Carlo simulation supported the sufficient size of the expected sample by indicating that cross-lagged models from the first to the fourth assessment can meaningfully test effects with a strength of* r* = 0.09, and mediation models can test indirect effects with a strength of *r* = 0.01 and a power of 0.95. For latent-growth models, the power exceeds 0.99 for the identification of predictors for the slope with an effect size of 0.15 [140]).

## Discussion

The PIER_YOUTH_-study is a prospective longitudinal study covering a period of 10 years and an age range from 6 to 20 years. The focus lies on the role of SR as a central resource for accomplishing different developmental tasks [1, 3]. The study enables to investigate the complex single and conjoint development of multiple SR skills from middle childhood to adolescence, thus including adolescence as a period that has not yet been sufficiently addressed in SR research [30] despite the numerous changes relevant to SR development (such as increasing autonomy or the development of more complex SR strategies). The study will also examine potential overlapping or differential prospective association patterns of single and conjoint SR skills with a broad range of outcome variables (e.g., prosocial behavior, delinquency, emotional problems, eating-related behaviors) that are highly relevant in this age range.

A particular strength of this study is the multi-method and multi-rater assessment of multiple SR sub-facets (e.g., delay of gratification, HRV, impulsivity, emotion regulation). Adoption of this multi-faceted battery facilitates complex data analyses on developmental trajectories of SR, other skills, or the interplay between SR sub-facets. Furthermore, the specific contribution of individual SR sub-facets to a broad range of adolescence-specific outcomes can be examined. This multifaceted assessment allows for not only examining developmental trajectories of SR and their influencing factors, but also the influence of SR on specific aspects (e.g., aggressiveness, delinquency) and their specific vs. generic relevance (i.e., whether the level and/or profile of SR sub-facets is predictive for different mental health problems). Consequently, implications for the development of theory-based and evidence-based, possibly outcome-specific SR interventions can be derived.

Some limitations also deserve attention: The PIER_YOUTH_ study includes a sample with an above-average educated family background in the German federal state Brandenburg, which is not representative of all German children and adolescents in that age group. Additionally, the survey had to strike a balance between the time burden on participants and the desired breadth of constructs. Therefore, we decided to use short versions of established instruments whenever possible. These short versions are time-efficient but have the potential disadvantage of lower reliabilities. Performing statistical analyses at the latent level can reduce this problem. Of note, due to the COVID-19 pandemic with access restrictions for researchers (e.g., at schools), the larger part of assessments at T4 are conduct online—in contrast to the previous face-to-face tests at schools. However, this also facilitates the recruitment of eligible individuals who no longer live in the region. Due to the extensive training of the test administrators, a high level of data quality can be ensured.

To sum up, there has been an increased interest to address questions on the development and effects of SR in a developmental framework covering an age range from middle childhood to adolescence. Prospective data over a longer time span are essential to foster our understanding of the emergence and role of self-regulation for a positive youth development. The PIER_YOUTH_-study aims to contribute to our understanding of the complex interplay of different SR sub-facets and their influence on child and adolescent development.

## Data Availability

Data generated in the course of the study will be available from the corresponding author on reasonable request and after a moratorium of five years following the completion of the study.

## Abbreviations

ABCL: Adult behavior checklist
AEBQ: Adult eating behavior questionnaire
ASR: Adult self-report
BART: Balloon analogue risk task
BE: Binge eating
BFI-S: Big five inventory-SOEP
BIS: Barratt impulsiveness scale
BMI: Body mass index
BRIEF: Behavior rating inventory of executive function
CBCL: Child behavior checklist
CEBQ: Children’s eating behavior questionnaire
CERQ: Cognitive emotion regulation questionnaire
CHQ: Child health questionnaire
CSBS-T: Children’s social behavior scale-teacher version
DCCS: Dimensional change card sorting task
DDT: German delay discounting test
DEBQ: Dutch eating behavior questionnaire for children
DISYPS-KJ: Diagnostik-system für psychische Störungen im Kindes-und Jugendalter; diagnostic system of mental disorders in children and adolescents
DTGA: Depressionstest für Kinder im Grundschulalter; depression test for children of elementary school age
EDE-Q: Eating disorder examination questionnaire
EF: Executive functions
ERQ: Emotion regulation questionnaire
FAI: Family adversity index
FASD: Fragebogen zum aussehensbezogenen druck; appearance-related social pressure questionnaire
FEEL-KJ: Fragebogen zur Erhebung der Emotionsregulation bei Kindern und Jugendlichen; questionnaire to elicit emotion regulation in children and adolescents
FIML: Full information maximum likelihood procedure
GAD-7: Generalized anxiety disorder assessment
GDPR: General data protection regulation
HRV: Heart rate variability
IDS: Intelligence and development scales
IRPA: Instrument for reactive and proactive aggression
JSI-CA: Justice sensitivity inventory for children and adolescents
LOCES: The loss of control over eating scale
MJF: Mannheimer Jugendlichen-Fragebogen; Mannheim adolescent questionnaire
O: Objective measures
OPD-SQS: Operationalised psychodynamic diagnostic-structural questionnaire short version
P: Parent-report
PB: Performance-based computer task
PHQ-8: Patient health questionnaire without item suicidality
PHQ-9: Patient health questionnaire
PIER: Potsdamer Internale Entwicklungs-Risiken; engl. Potsdam intrapersonal developmental risk study
POTS: Perception of teasing scale
QEWP: Questionnaire of eating and weight patterns
S: Self-report
SATAQ‐IV-R: Sociocultural attitudes towards appearance questionnaire-IV revised
SDQ: Strengths and difficulties questionnaire
SEA-K: Skala zur Erfassung von Testverfälschung durch positive Selbstdarstellung und sozial erwünschte Antworttendenzen; scale for detecting test manipulation through faking good and social desirability bias
SES: Socioeconomic status
SIP: Social information processing
SPSS: Statistical package for the social sciences
SR: Self-regulation
SRS: Self-regulation scale
SSIPA: Social information processing in adolescence
T: Teacher-report
T1/T2/T3/T4: First/second/third/fourth measurement point
TMCQ: Temperament in middle childhood questionnaire
UPPS: UPPS impulsive behavior scale with subscales urgency, premeditation, perseverance, sensation seeking, and positive urgency
WAIS-IV: Wechsler adult intelligence scale-fourth edition
WBIS-C: Weight bias internalization scale for children
WISC-IV: Wechsler intelligence scale for children-fourth edition
YSR: Youth self-report
ZN-R: Zahlen nachsprechen-rückwärts; German version of the digit span backwards subtest oft he WISC-IV
ZST: Zahlensymboltest; symbol digit modalities test (SDMT)
